# EASY-C: Extraction and Analysis of Small Yeast Chromosomes—A rapid and universal platform for recovering artificial mini-chromosomes from synthetic Sc2.0 yeast and large plasmids from *Saccharomyces cerevisiae* and nonconventional yeast species

**DOI:** 10.1093/synbio/ysag002

**Published:** 2026-01-21

**Authors:** Reem Swidah, Marco Monti, Daniela Delneri

**Affiliations:** Manchester Institute of Biotechnology, the University of Manchester, 131 Princess Street, M1 7DN Manchester, United Kingdom; Manchester Institute of Biotechnology, the University of Manchester, 131 Princess Street, M1 7DN Manchester, United Kingdom; San Raffaele Telethon Institute for Gene Therapy, IRCCS San Raffaele Scientific Institute, Milan, 20132, Italy; Manchester Institute of Biotechnology, the University of Manchester, 131 Princess Street, M1 7DN Manchester, United Kingdom

**Keywords:** artificial synthetic chromosome, high-/low-copy-number plasmid, Sc2.0 synthetic yeast, nonconventional yeast, nanopore sequencing

## Abstract

The protocol for Extraction and Analysis of Small Yeast-Chromosomes (EASY-C) is a transformative, rapid, cost-effective, and user-friendly method designed for the efficient isolation of artificial synthetic mini-chromosomes (~42–52 kb) and large plasmids (~12 kb) from *Saccharomyces cerevisiae* wild-type and synthetic yeast strains (Sc2.0), and a range of nonconventional yeast (NCY) species. In this two-step workflow, the DNA from yeast is first extracted and transferred into bacteria, and then the circular DNA is recovered from the bacteria and subjected to downstream analysis, including long-read sequencing. The EASY-C protocol operates at small volumes (~1 mL) and requires less than 2.5 hours, allowing the use of standard commercial plasmid purification kits for bacterial plasmids. Under the tested conditions, the EASY-C methodology yielded clean DNA that could be digested and linearized prior to sequencing, resulting in a higher number of high-quality reads (~2000). The EASY-C protocol worked successfully for the extraction of a variety of constructs, including low-copy centromeric vectors (CEN/ARS), high-copy plasmids (pan/ARS), and artificial mini-chromosomes harbouring (CEN/ARS). It is also applicable to a variety of yeast species, including NCY such as *Starmerella* sp.*, Maudiozyma* sp.*, and Kazachstania* sp*.* Thanks to its precision, robustness, and simplicity, EASY-C equips researchers with a powerful, time-saving tool and cost-effective approach to accelerate the validation of a wide array of synthetic genetic and metabolic constructs engineered *in vivo* across diverse yeast species.

## Graphical Abstract

**Graphical Abstract:**
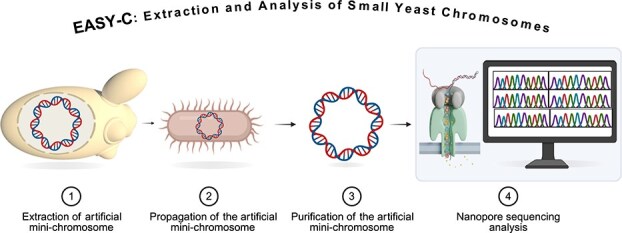

## Introduction

**Figure 1:**
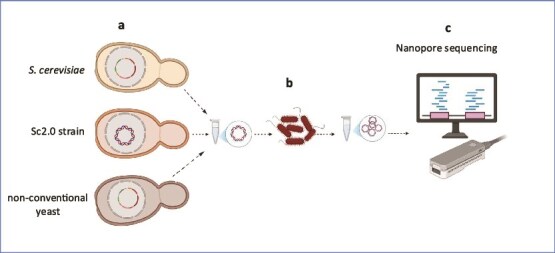
The EASY-C protocol: A streamlined and scalable workflow for circular DNA recovery and high-quality sequencing. Schematic overview of the EASY-C protocol. (a) Different yeast species, including a wild-type *S. cerevisiae* strain, a Sc2.0 synthetic strain, and NCY yeasts carrying a circular artificial mini-chromosome or large plasmids are grown in selective media. (b) Plasmids or artificial mini-chromosomes are extracted from yeast cells and transformed into *E. coli* for propagation. (c) Following linearization, the DNA is subjected to long-read sequencing (Oxford Nanopore). This approach consistently yields high-quality long-read data with robust coverage.

The yeast *Saccharomyces cerevisiae* has long been celebrated as a powerful model microorganism in systems and synthetic biology. One of its most remarkable features is the efficiency of its homologous recombination (HR) machinery, which enables the construction and engineering of large synthetic genetic constructs in vivo in yeast in a single step [1, 2]. Leveraging various types of circular plasmids as destination vectors, researchers have successfully reconstituted complex metabolic pathways and even built synthetic chromosomes—most notably within the groundbreaking Sc2.0 project [3]. This initiative has redefined synthetic genomics, pushing the boundaries of what is possible in genome design and manipulation. *Saccharomyces cerevisiae* stands out not only as a genetic model but also as a versatile cell factory with extensive applications in biotechnology, including the production of biofuels, vaccines, pharmaceuticals, and natural products [4]. The Sc2.0 synthetic yeast project involves the *in silico* design and bottom-up synthesis of a eukaryotic genome [5]. With the Sc2.0 project approaching its final stages, the goal is to engineer a fully synthetic S. cerevisiae strain exhibiting unprecedented genome-wide evolutionary capacity [3]. Across the genome, thousands of modifications were introduced, including the deletion of mobile elements and introns, relocation of transfer ribonucleic acid (tRNA) genes, and conversion of TAG stop codons to TAA, thereby reserving TAG for potential encoding of nonstandard amino acids [5]. A genomic watermarking system, PCRtag, was implemented by synonymously recoding short segments of open reading frames (ORFs). This system allows primer pairs to selectively detect either synthetic or wild-type (WT) chromosomes by PCR, enabling distinction between synthetic and native genomic content [6]. Furthermore, palindromic loxPsym sites were inserted into the 3′ untranslated region (UTR) of each nonessential gene to facilitate Cre recombinase–mediated genome-wide rearrangements through the SCRaMbLE (Synthetic Chromosome Rearrangement and Modification by LoxP-mediated Evolution) system. Through the SCRaMbLE system, this synthetic yeast can undergo rapid, large-scale genomic diversification, enabling the development of tailor-made strains with enhanced phenotypes [7]. These modifications were designed to enhance genome stability, enable the generation of diverse phenotypes, and address key biological questions [8–15]. While synteny of protein-coding genes is preserved during assembly, the synteny of tRNA genes is intentionally disrupted in the synthetic chromosomes. The SCRaMbLE system additionally allows controlled reshuffling of the protein-coding gene order following initial chromosome synthesis. To date, six complete synthetic chromosomes (synII, synIII, synV, synVI, synX, and synXII) and one synthetic chromosome arm (synIX right arm)—collectively referred to as ‘6.5 chromosomes’—have been constructed. A notable example includes the evolution of a super hygromycin-B-resistant strain, potentially opening new avenues for antibiotic production [16]. In parallel, attention is turning to nonconventional yeasts (NCYs), which offer broader physiological diversity than *S. cerevisiae*, and could be developed as production hosts for green chemicals. Species such as *Starmerella bombicola*, *Starmerella batistae*, *Kazachstania naganishii*, *Kazachstania aerobia*, and *Maudiozyma bulderi* exhibit unique biotechnological traits. Such characteristics make them promising candidates for the production of organic acids, acetate esters, sophorolipids, palm oil–derived sophorolipids, and other specialty chemicals [17–20]. Isolating plasmids from yeast is a critical step in molecular genetics, enabling key downstream applications such as restriction digest analysis, Polymerase Chain Reaction (PCR) analysis, DNA sequencing, and transformation into bacterial or other yeast hosts. However, traditional plasmid recovery methods are often considered a major bottleneck due to their typically low yields and labour-intensive protocols [21]. Common extraction techniques—including mechanical disruption [22], chemical treatment [23], enzymatic digestion using lytic enzymes [23, 24], and phenol–chloroform extraction—often compromise plasmid purity and quality, complicating further chemical and physical analysis. Residual contaminants such as phenol, cellular proteins, and other macromolecules can interfere with physical and chemical assays, reducing the reliability of experimental outcomes. In our previous work, we optimized an extraction protocol capable of recovering an artificial tRNA-based neochromosome (~250 kb) from Sc2.0 synthetic yeast and successfully transplanting it into various Sc2.0-derived synthetic strains [12]. Although this method preserved the integrity of large circular synthetic constructs, it required the preparation of several different buffers and was time-consuming. Similarly, Singh *et al.* (2002) proposed a method to extract plasmids from yeast using 1-l cultures and lyticase digestion, yet this approach also relies on large reagent volumes and remains resource-intensive. To overcome these challenges, we have developed a new Extraction and Analysis of Small Yeast Chromosomes (EASY-C) protocol, an efficient, eco-friendly, and time-saving approach for plasmid/mini [5] artificial chromosome isolation directly from just 1 ml of fresh overnight yeast culture (Fig. 1). This streamlined protocol utilizes the same reagent volumes as standard bacterial plasmid extraction kits (e.g. Qiagen), eliminating the need for large-scale cultures and reducing reagent consumption. As proof of principle for the EASY-C protocol and to be sequence-agnostic, we employed different types of constructs, such as both low-copy-number (CEN/ARS) and high-copy-number (pan/ARS) plasmids of varying lengths (6–12 kb) and guanine and cytosine (GC) content ≤ 31%, as well as a synthetic mini-chromosome (42 kb, 37% GC content). This new method enables the recovery of conventional low- and high-copy-number plasmids as well as synthetic mini-chromosomes of length ≤ 52 kb. In a wide range of NCYs, the EASY-C protocol also allows the recovery of large-sized plasmids (~12 kb) with a high copy number harbouring a pan/ARS origin of replication. By simplifying and accelerating plasmid recovery, this enhanced protocol empowers researchers to conduct high-quality genetic analysis while minimizing time, cost, and environmental impact—paving the way for more accessible and sustainable synthetic biology research using both *S. cerevisiae* WT, Sc2.0 synthetic yeast strains, and NCYs.

## Material and methods

### Strains, plasmids and media

The laboratory strains used in this study included *Saccharomyces cerevisiae* WT BY4741 and the Sc2.0 semisynthetic yeast synIII strain harbouring a synthetic chromosome, obtained from Yizhi Cai, Manchester Institute of Biotechnology, University of Manchester, Manchester, United Kingdom. NCY species, including *S. bombicola, S. batistae, M. bulderi CBS8639, K. naganishi*, and *K. aerobia* were obtained from Daniela Delneri, Manchester Institute of Biotechnology, University of Manchester, Manchester, United Kingdom. All the plasmids used in this study were shuttle vectors capable of replication in both *Escherichia coli* and yeast. For *S. cerevisiae* (WT and Sc2.0 strains), low-copy-number centromeric vectors (CEN/ARS), derived from the pRS vector backbone, were used [25] and the CRISPR/Cas9 expression creepy system, derived from Jef D. Boeke, Institute for Systems Genetics, NYU Grossman School of Medicine, New York University, New York, NY, United States [26]*.* For NCYs, the pUDbzB-41 vector, which harbours a CRISPR/Cas9 cassette and a compatible origin of replication (pan/ARS), enabled propagation and maintenance in these species as a high-copy-number plasmid. The pUDbzB-41 vector was optioned from Paola Branduardi—Department of Biotechnology and Biosciences, University of Milano-Bicocca, Piazza della Scienza 2, 20126 Milano, Italy [27]—and a pRS vector harbouring a pan/ARS element was constructed for this study. The media used in all the yeast experiments was standardized YPD medium (10 g/l yeast extract, 20 g/l peptone, and 20 g/l dextrose), supplemented with G418 antibiotic (Geneticin; Thermo Fisher Scientific, United States) which serves as the selective marker for the plasmid or minimal media lacking uracil to maintain plasmid selection. For bacterial selection of transformants carrying the plasmid or artificial mini-chromosome, standard Luria broth (LB) medium supplemented with carbenicillin (Carbenicillin; Thermo Fisher Scientific; USA) was used.

### EASY-C procedure for synthetic mini-chromosome and plasmid

Yeast cultures (1 ml) carrying circular synthetic artificial chromosomes on the pRS vector, or plasmids of varying lengths and copy numbers, were grown overnight to stationary phase (~10 OD_600_/ml) in YPD medium (10 g/L yeast extract, 20 g/L peptone, 20 g/L dextrose) supplemented with G418 or in uracil-deficient minimal media to maintain plasmid or mini-chromosome selection. The extraction efficiency was verified using the pRS vector as a positive control. A final concentration of 400 μg/ml G418 was used for wild-type or Sc2.0 synthetic S. cerevisiae strains, while appropriate concentrations were applied for NCY strains. Cultures were incubated at the optimal temperature: 30°C for *S. cerevisiae* WT and Sc2.0 strains and 25°C or 30°C for NCY strains. Following incubation, cells were harvested by centrifugation at 4000 rpm for 5 minutes at room temperature. The resulting pellet was resuspended in 250 μl of Buffer P1 (Qiagen Plasmid Miniprep Kit; Qiagen, Valencia, CA) by pipetting up and down gently. To induce partial spheroplast formation, 100 μl of lyticase solution (2000 units/ml; ICN Biomedicals, Aurora, OH) prepared in 1.2 M sorbitol and 0.1 M sodium phosphate buffer (pH 7.4), containing 5 mg/ml *Arthrobacter luteus* lyticase, was added. The mixture was incubated at 37°C for 60–90 minutes to digest the cell wall. Specifically, the concentration of lyticase or the incubation time can be increased to enhance cell wall digestion. The DNA extracted from 1 ml of an overnight culture in stationary phase with an OD_600_ = ~10 produced sufficient transformants for both plasmid and artificial mini-chromosome extraction. Stationary-phase *S. cerevisiae* cells are more difficult to lyse than exponentially growing cells due to a thicker, more highly cross-linked cell wall (including increased disulphide bridges), which reduces permeability and enhances mechanical robustness [28]; hence, we tested the Easy-C protocol in the worst case scenario and we showed that it can efficiently extracts plasmids or artificial mini-chromosomes. Mechanical disruption with beads was avoided to minimize shearing of large circular DNA molecules, such as artificial mini-chromosomes. Next, 250 μl of Buffer P2 was added to lyse the cells, and the tube was gently inverted several times and incubated at room temperature for 10 minutes. During this step, cell lysis led to DNA release and an increase in viscosity of the solution. Subsequently, 350 μl of Buffer N3 was added to the lysate, followed by gentle mixing and incubation on ice for 30–40 minutes. The lysate was then centrifuged at 13 000 rpm for 10 minutes at 4°C. To facilitate DNA binding, 800 μl of the clear supernatant was applied to Qiagen QIAprep Spin Miniprep columns in small aliquots, and then centrifugation was conducted at 13 000 rpm for 1 minute at room temperature (QIAprep Spin Miniprep Kit, Qiagen, Cat. No. 27104/27106). The columns were washed with 500 μl of Buffer PB and then centrifuged at 13 000 rpm for 1 minute at room temperature. A second wash was performed using 750 μl of Buffer PE, followed by centrifugation at the same speed and temperature. After removing the flow-through, an additional 1-minute spin was performed to eliminate residual buffer. The synthetic mini-chromosome or plasmid DNA was eluted by adding 50 μl of elution buffer (Buffer EB) or endonuclease-free water to each column, incubated at 22°C for 5 minutes, and centrifuged at 13 000 rpm for 5 minutes at room temperature. The DNA concentration derived from the yeast was measured using a Nanodrop spectrophotometer, which yielded concentrations between 30 and 100 ng/μl depending on the quality of the extraction and plasmid copy number. To improve extraction outcomes, we recommend gentle handling throughout the process—mixing by inversion rather than vortexing and using wide-bore pipette tips to minimize shearing. Synthetic mini-chromosomes maintained on centromeric plasmids containing CEN/ARS were typically present at low copy numbers (1–3 copies per cell), while plasmids containing pan/ARS elements were present in high copy numbers (often >20–70 copies per cell). Plasmid extractions from the WT yeast strain containing the pRS vector were carried out 10 times for the WT (i.e. 10 replicates). For the synthetic yeast strain to extract the artificial mini-chromosome, two biological replicas were carried out (i.e. independent extractions), which yielded similar DNA concentrations and purity. For NCY strains, one biological replica was carried out to recover the plasmid from yeast and three biological replicas of plasmid recovered from *E. coli* were sequenced. One representative example is provided in Table S1.

### Plasmid constructions

The plasmids were assembled *in vivo* using the standard homologous-recombination machinery of yeast, with 60-bp homology regions flanking each genetic part [29].

### Plasmid extraction from bacteria using the QIAprep Spin Miniprep Kit

Plasmid DNA was purified from bacterial cultures using the QIAprep Spin Miniprep Kit (QIAGEN, Cat. No. 27104/27106) according to the manufacturer’s instructions (QIAprep Miniprep Handbook). Briefly, 1–5 ml of overnight bacterial culture grown in LB medium with ampicillin at 37°C was harvested. Cells were resuspended in 250 μl of Buffer P1, followed by addition of 250 μl of Buffer P2 and gentle mixing for 5 minutes. Subsequently, 350 μl of Buffer N3 was added, and the lysate was centrifuged at maximum speed (13 000 rpm) for 10 minutes. Approximately 800 μl of the supernatant was transferred to a spin column and centrifuged; the flowthrough was discarded. Next, 500 μl of Buffer PB was added to the column and centrifuged; the flowthrough was discarded. The column was washed with 500 μl of Buffer PE and centrifuged; the flowthrough was discarded. The column was spun again to remove any residual wash buffer. The spin column was then transferred to a new microcentrifuge tube, and DNA was eluted with water by incubating for 5 minutes at room temperature, followed by centrifugation. Purified plasmid DNA was quantified using a NanoDrop spectrophotometer. The DNA concentration for the mini-chromosome in bacteria was normal, ranging from 100 to 200 ng/μl.

### High-molecular-weight plasmid and genomic DNA extraction

High-molecular-weight plasmid DNA and genomic DNA were extracted from yeast using the NucleoBond™ HMW DNA Kit (Macherey-Nagel, Cat. No. 740180), following the manufacturer’s protocol optimized for yeast samples. RNase was used during the extraction process to remove residual RNA, which can negatively impact nanopore sequencing quality when it is extracted directly from yeast.

### Bacterial transformation

To perform bacterial transformation, chemically competent *E. coli* DH5α cells were used. For each transformation, one vial of 50 μl competent cells was thawed on ice. Subsequently, 5 μl of plasmid DNA (ranging from 30 to 100 ng/μl) was added to the cells and gently mixed without pipetting up and down. As a positive control, 1 μl (10 pg) of pUC19 plasmid DNA was added to a separate vial of competent cells and mixed gently. The mixtures were incubated on ice for 30 minutes, and then heat shock was applied at 42°C for 45 s without shaking. Immediately after the heat shock, the vials were placed back on ice for 5 minutes. Each transformation mixture was then supplemented with 950 μl of prewarmed S.O.C. medium and incubated at 37°C for 1 hour in a shaking incubator at 225 rpm. After incubation, the cells were centrifuged at 4000 rpm for 5 minutes at room temperature, and 900 μl of the supernatant was removed to concentrate the cells. A total of 100 μl of each transformation mixture was spread onto prewarmed selective agar LB + carbenicillin plates. The plates were incubated overnight at 37°C, and the transformation efficiency was verified using the pUC19 positive control. DH5α Mix & Go competent cells were also used for bacterial transformation when the plasmid size was relatively small (< 10 kb), following the manufacturer’s instructions (Zymo Research, Orange, CA). However, the competent cells were prepared at a higher concentration—three times more concentrated than the manufacturer’s recommendation (Mix & Go! Competent Cells—DH5 Alpha, Cat. No. T3007/T3009, Zymo Research, United States).

### Restriction digest analysis

Plasmid DNA was analysed by restriction enzyme digestion using New England Biolabs (NEB) enzymes. Appropriate restriction enzyme pairs were selected using the NEB Double Digest Finder tool to determine the optimal NEBuffer, recommended incubation temperature, and whether bovine serum albumin (BSA) supplementation was necessary. For each reaction, up to 1000 ng of plasmid DNA (typically 3–5 μl of miniprep) was mixed with 1 μl of 10× NEBuffer and 0.5 μl each of two selected restriction enzymes (1 Fast Digest unit per microlitre). When recommended, 1 μl of 10× BSA was also included. The reaction mixture was brought to a total volume of 10 μl with nuclease-free distilled water. Reactions were incubated for at least 1 hour or left overnight at the specified temperature in a metal heating block or water bath. Following digestion, 2.5 μl of 5× DNA loading dye was added to each sample, and the digested products were analysed by agarose gel electrophoresis on 1% agarose gel.

### Long-read nanopore sequencing

The aim of nanopore sequencing was to detect potential chromosome rearrangements (CRs), single nucleotide polymorphisms (SNPs), or insertions and deletions (indels) in the extracted chromosome. DNA purity and integrity were assessed prior to sequencing using standard agarose gel electrophoresis, a NanoDrop™ 2000 Spectrophotometer (Thermo Fisher Scientific, Waltham, MA, United States), and a Qubit 4 Fluorometer (Thermo Fisher Scientific, Waltham, MA, United States) with dsDNA BR reagents to ensure high-quality input DNA. The circular mini-chromosome was linearized using any restriction enzyme that cuts once within the backbone. The restriction digest product was purified using the QIAquick PCR Purification Kit (Qiagen, Hilden, Germany) according to the manufacturer’s instructions, and then subjected to nanopore sequencing analysis.

### Library preparation and nanopore sequencing

Library preparation was performed using the SQK-LSK109 Ligation Sequencing Kit and either the Native Barcoding Kit EXP-NDB104 or EXP-NDB114, following the manufacturer’s instructions (Oxford Nanopore Technologies, Oxford, United Kingdom). The protocol was modified by increasing the starting DNA input to ~2 μg without mechanical shearing to achieve sufficient molarity and maintain long-read integrity. The sequencing was conducted using a MinION Mk1B device equipped with a Flongle flow cell (R9.4.1, FLO-FLG001), with a total runtime of 24 hours. The target sequencing depth was >20× coverage relative to the reference genome.

### Nanopore data analysis

Base calling of the raw signals was conducted locally using Guppy v5.0.11 (Oxford Nanopore Technologies). Read quality metrics were evaluated using NanoPlot v1.35.5 [30]. Alignment to the reference genome was performed with either Minimap2 v2.20-r1061 [31] or NGM-LR v0.2.7 [32]. Variant calling, including detection of structural variants, was done using Sniffles v1.0.12 [32], while *de novo* genome assembly was carried out using Canu v2.1.1 [33]. In addition to the in-house Oxford Nanopore sequencing performed on the artificial mini-chromosome, whole-plasmid nanopore sequencing was outsourced to Plasmidsaurus (United Kingdom) using DNA extracted from bacteria.

## Result and discussion

### Extraction efficiency using the EASY-C protocol

We applied the EASY-C protocol to a range of yeast strains, including a WT strain, the Sc2.0 synthetic strain, and several nonconventional species. Using this method, we successfully extracted plasmids of varying sizes, distinct replication mechanisms, and an artificial mini-chromosome relevant to the synthetic biology community. Among the strains tested, *K. naganishii* yielded the highest number of colonies in NCY medium, whereas *M. bubodieri CBS8639* produced the lowest. As expected, circular DNA recovered directly from yeast may contain sheared plasmid species and residual genomic DNA. Although A260/A280 ratios typically remain within the expected range of 1.8–2.0, the colony forming units (CFU) per microgram of DNA is often lower than that obtained using pure, supercoiled plasmid DNA isolated from bacteria [34]. All the information for the constructs is in Table S1.

### Plasmid extraction efficiency using the EASY-C protocol in the WT *Saccharomyces cerevisiae* strain

**Figure 2:**
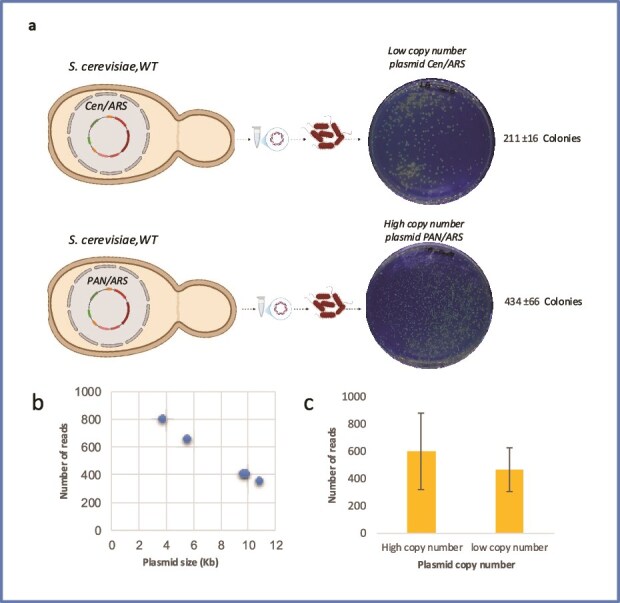
Plasmid extraction efficiency using the EASY-C protocol in wild-type *S. cerevisiae* strains carrying high- and low-copy plasmids. (a) Plasmid recovery in *E. coli* from wild-type *S. cerevisiae* harbouring either a low-copy-number plasmid (CEN/ARS, top) or a high-copy-number plasmid (pan/ARS, bottom). (b) Number of reads obtained via nanopore sequencing for plasmids of different sizes. (c) Number of reads obtained via nanopore sequencing for plasmids with different copy numbers: the high-copy-number plasmid (10.3 kb) and the low-copy-number plasmid (~6 kb). Error bars are ± SEM from three biological replicates.

Firstly, we tried our extraction method on the WT yeast *S. cerevisiae* (Fig. 2). We assessed the extraction efficiency of a large CRISPR/Cas9 expression vector (11 603 bp) containing a CEN/ARS origin of replication—typically maintained at low copy numbers (1–3 copies per cell). In parallel, we analysed the same CRISPR/Cas9 expression vector where the CEN/ARS was swapped with a pan/ARS-based episomal vector (10 046 bp), well known for its ability to replicate across a broad range of conventional and nonconventional yeast species, and the ability to achieve a high copy number, ranging from 6 to 50 copies per cell depending on specific ARS elements and the yeast strain used [35]. As a main control, we used a smaller pRS centromeric plasmid (<5000 bp), which includes a yeast auxotrophic marker and serves as a shuttle vector, capable of replication in both yeast and *E. coli*. To extract the plasmids, we cultivated 1 ml overnight yeast cultures in selection media (either YPD with G418 or SD minimal media lacking uracil (URA)) at 30°C. Cells were harvested and processed using the EASY-C protocol as described in the Materials and Methods section. Yields ranged from 30 to 100 ng/μl in a 50 μl elution volume, depending on the plasmid copy number and the quality of the extraction. Overall, this concentration is not typically sufficient to perform downstream experiments, such as enzymatic digestions and sequencing. This is also because the plasmid extraction might contain some genomic DNA contamination and has sheared plasmids (a problem that is more severe for larger vectors). Next, to improve the plasmid quantity, we used ~300 ng of freshly extracted plasmid DNA from yeast in 50 μl of competent *E. coli* DH5α cells; as expected, the transformation efficiency reflected the plasmid copy number: given the same DNA concentration and plasmid sizes, we obtained twice as many colonies from the high-copy-number plasmid compared to the CEN/ARS vector (Fig. S1). These results suggest that a low plasmid copy number is indeed a limiting factor in transformation success, especially when working with centromeric vectors. Transformant bacterial colonies were grown on selective media. Plasmids were then purified using a miniprep, yielding a high concentration of pure plasmids (~500–700 ng/μl). We could successfully carry out double digestion analysis to confirm the size of the constructs. The Easy-C protocol was optimized based on the method described by Singh and Weil [21], who used plasmids from 1 l of yeast culture, subsequently propagated them in *E. coli*, and used large volumes of Qiagen reagents (5 ml Buffer P1, 5 ml lyticase solution, and 10 ml Buffer P2). In contrast, the Easy-C protocol substantially scales down and adapts this approach for small-scale workflows. The culture volume was reduced to 1 ml of yeast, and reagent volumes were optimized accordingly, namely 100 μl lyticase (100 U) and 250 μl each of buffers P1 and P2. This protocol produces a 1000-fold reduction in scale and markedly reduces reagent consumption, lab disposables, and the overall experimental running cost. We were able to retrieve sufficient DNA for bacterial transformation and efficiently recovered circular DNA suitable for nanopore sequencing. Chin-Sang [36] uses zymolyase for enzymatic cell-wall digestion, followed by chemical lysis (1 ml 10% SDS and 200 μl 10 N NaOH in 10 ml H₂O) and neutralization with 4 M KOAc (pH 5.5). Although conceptually similar to our simplified workflow for isolating standard plasmids from yeast, this method does not assess the recovery of synthetic chromosomes from engineered yeast strains or the extraction of large plasmids from NCYs.

### Mini-Chromosome recovery in Sc2.0 synthetic strain via the EASY-C protocol

The EASY-C protocol is also suitable for recovering artificial synthetic circular mini-chromosomes ranging from 42 to 52 kb in size from the semisynthetic *S. cerevisiae* Sc2.0 strain (synIII). These artificial mini-chromosomes were engineered on a centromeric vector, present at a low copy number (1–3 copies per cell), making their extraction particularly challenging due to both their size and limited abundance. To provide a benchmark, we used a standard pRS centromeric plasmid (<5000 bp) as a positive control. Yeast cultures were grown in 1 ml of selective media—either YPD with G418 or SD minimal media lacking uracil—at 30°C overnight. Cells were then harvested and processed using the EASY-C protocol as detailed in the Materials and Methods section. There are technical challenges associated with recovering large circular DNA elements (>42–52 kb), because of their vulnerability to shearing during extraction and transformation. We found that using wider pipette tips helps to recover intact artificial mini-chromosomes by reducing DNA shearing. Accordingly, mechanical disruption with beads was avoided to minimize damage to large circular DNA molecules, such as artificial mini-chromosomes. One of the major challenges in recovering large plasmids or synthetic chromosomes is that the likelihood of DNA shearing increases with the size of the plasmid or circular chromosome. Therefore, in the Easy-C protocol, enzymatic digestion of the yeast cell wall with lyticase was used. Specifically, the concentration of lyticase or the incubation time can be adjusted to enhance cell wall digestion.

**Figure 3:**
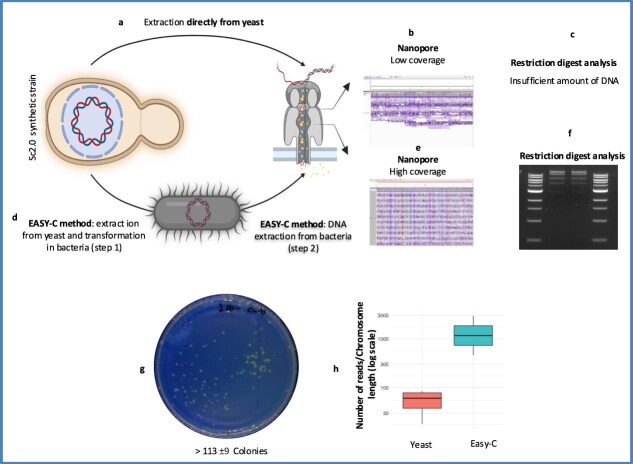
Enhanced data quality and coverage from *E. coli*-derived artificial mini-chromosome compared to direct yeast sequencing. (a) The artificial mini-chromosome was directly extracted from yeast using the NucleoBond™ HMW DNA Kit and subjected to Oxford Nanopore sequencing. (b) Sequencing data from the yeast-extracted mini-chromosome showed low read lengths and poor coverage. (c) Restriction digest analysis could not be performed on the mini-chromosome extracted directly from the Sc2.0 synthetic yeast strain due to insufficient DNA yield. (d) The EASY-C protocol involves step 1: extraction of the mini-chromosome from yeast and transformation into *E. coli*, followed by step 2: extraction of the mini-chromosome from bacteria and subjected to Oxford Nanopore sequencing. (e) Sequencing data from the *E. coli*–propagated mini-chromosome displayed higher-quality, longer reads, and substantially improved coverage after linearization. (f) Restriction digest analysis confirmed correct assembly of the mini-chromosome after propagation in *E. coli*. Eight biological replicates were used for DNA extraction and digestion from *E. coli*, all yielding high DNA amounts. (g) Plasmid recovery in *E. coli* from the Sc2.0 strain harbouring a low-copy synthetic artificial mini-chromosome (CEN/ARS). (h) Nanopore sequencing data showing the number of reads obtained using the standard protocol (direct yeast extraction) and the EASY-C protocol. Two biological replicates from direct yeast extraction were sequenced from yeast using nanopore sequencing. Two biological replicates from bacteria were processed using the EASY-C protocol. Each biological replicate was sequenced twice (i.e., two technical replicates) using nanopore sequencing.

The yield of the mini-chromosome extraction from synthetic yeast cells ranged from 25 to 30 ng/μl. To improve the plasmid quantity, we used ~300 ng of freshly extracted plasmid DNA from yeast to transform *E. coli* DH5α cells. We successfully obtained ~41–100 bacterial transformants with low-copy-number artificial mini-chromosomes (Fig. 3). Selected bacterial transformants were cultured, and the mini-chromosome was purified, yielding ~200–300 ng/μl. The mini-chromosome was analysed through double restriction enzyme digestion.

### EASY-C protocol for medium- and small-sized plasmid recovery in NCYs

**Figure 4:**
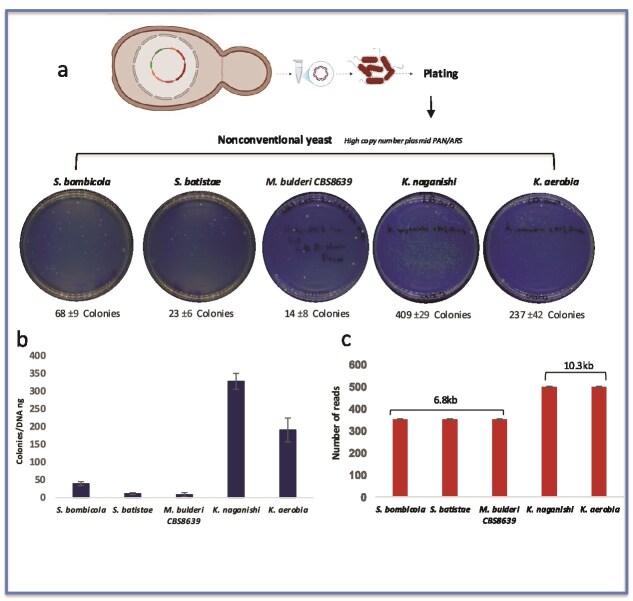
The EASY-C protocol is effective across a wide range of nonconventional yeast species that are genomically highly diverged from *S. cerevisiae*. (a) Number of *E. coli* colonies obtained after applying the EASY-C protocol to high-copy plasmids (pan/ARS) extracted from various NCY species. (b) Number of *E. coli* colonies obtained after transformation, normalized to DNA concentration. Error bars are ± SEM from three replicates. (c) Number of reads obtained from bacteria-derived plasmids of different sizes. Error bars are ± SEM from three biological replicates.

We extended the application of the EASY-C protocol beyond *S. cerevisiae*, demonstrating its potential in recovering medium-sized plasmids from a diverse set of NCYs, including *S. bombicola, S. batistae, M. bulderi, K. naganishi*, and *K. aerobia* (Fig. 4). Each of these strains harboured a pan/ARS-based episomal CRISPR/Cas9 expression vector (~10 046 bp), designed for broad-host-range replication across multiple yeast species [35]. These vectors can achieve high copy numbers—typically ranging from 20 to 70 copies per cell—depending on the ARS sequence and the host strain, and are thus ideal tools for genome editing in emerging yeast platforms. A pRS vector (~6,300 bp) engineered with pan/ARS, capable of propagating in NCYs, was also used in this study. Yeast cultures were grown in 1 ml of selective media (YPD supplemented with G418) at each strain’s optimal growth temperature and incubated overnight. For all the NCYs, we used only plasmids with high copy numbers. Using the EASY-C protocol, we obtained 25–35 ng/μl of a small-plasmid-bearing pan/ARS (6300 bp), which yielded 200–350 bacterial colonies in *K. naganishii* and *K. aerobia*. A few transformants were inoculated in selective media and the plasmids were extracted using the classical miniprep method. This resulted in a higher concentration of pure plasmids (~300–450 ng/μl), sufficient for restriction digest analysis. Similarly, we obtained 25–35 ng/μl to extract a medium-sized plasmid (10 300 bp) from *S. bombicola*, *S. batistae*, and *M. bulderi CBS8639*, which yielded only 5–12 colonies (here, the plasmid size significantly impacts the number of bacterial colonies obtained from this group of yeasts; Fig. 4). Using the EASY-C protocol (i.e. transforming the plasmids in *E. coli*), the plasmids extracted had a concentration of ~100–400 ng/μl, sufficient for restriction digest analysis.

### Comparison of Easy-C with commercial yeast plasmid extraction kits

Commercial yeast plasmid extraction kits (e.g. Qiagen, Zymo, Promega, and Thermo Fisher) are not optimized for recovering large circular DNA structures, such as synthetic artificial mini-chromosomes. Easy-C overcomes these limitations through several methodological improvements. It employs gentle enzymatic cell-wall digestion with lyticase, thereby avoiding bead-beating steps that can shear large circular DNA—including ~52-kb synthetic artificial mini-chromosomes—and preserves chromosome integrity through wide-bore pipetting and minimal mechanical handling. While commercial kits efficiently recover small plasmids, they consistently fail to isolate intact Sc2.0 synthetic artificial mini-chromosomes. In contrast, Easy-C is both highly cost-effective and time-efficient, costing approximately £0.96 per preparation and requiring <2.5 hours. By comparison, high-molecular-weight column-based kits (e.g. the NucleoBond™ HMW DNA Kit) cost approximately £22 per preparation, require 9–12 hours, and often yield fewer usable long-read sequencing reads due to copurified contaminants with genomic DNA and RNA. In the Easy-C protocol, we have scaled down the initial culture volume to 1 ml and provided sufficient DNA for bacterial transformation and nanopore sequencing. This improves upon earlier large-scale plasmid extraction methods that require substantially larger reagent volumes [21], offering a compact and cost-effective alternative for large circular synthetic chromosomes. By contrast, the Zymo kit fails to recover intact Sc2.0 artificial mini-chromosomes under equivalent conditions. Cost comparisons further highlight Easy-C’s advantages. Zymo Yeast Plasmid Miniprep I (approximately £1.44 per prep) remains more expensive than Easy-C (approximately £0.96 per prep), and high-molecular-weight, column-based extraction kits commonly used in synthetic biology labs (approximately £22 per prep) are substantially less economical (also requiring 9–12 hours to elute the DNA; and DNA and RNA contaminants are not removed). Crucially, Easy C allows us to use the Plasmidsaurus service for nanopore sequencing (approximately £15 and £30 for small and large plasmids, respectively, per sample), which is cheaper than the in-house Oxford Nanopore sequencing (approximately £80 per sample). Overall, Easy-C represents an optimized, miniaturized, and cost-efficient adaptation of existing methods, specifically designed to recover intact large circular synthetic chromosomes or plasmids from minimal yeast culture volumes for long-read sequencing. The total cost depends on both the plasmid extraction method and the sequencing approach used (Tables S2 and S3).

## DNA quality verified via long-read sequencing

### Plasmids extracted from the WT *S. cerevisiae*

We performed long-read sequencing via Oxford Nanopore (Plasmidsaurus, UK) on 800 ng of pure plasmid derived from bacteria, and we performed mapping of the reads against the reference genome and *de novo* assembly of the reads to validate the absence of any recombination or any mutations (SNPs or indels). The sequence data were of high quality, and we obtained an average coverage of 450× (Fig. 2). We did not find any evidence of structural rearrangements and detected no SNP mutations in 95% of the candidates tested, indicating that ~95% of the plasmids retain an identical sequence to the plasmid assembled *in vivo* in yeast. Within our dataset, for plasmids derived from bacteria, only 1 out of 20 samples contained a point mutation.

Our findings reinforce the value of EASY-C as a powerful, accessible method for plasmid recovery and downstream analysis.

### Mini-Chromosome extracted from the Sc2.0 semisynthetic strain

We performed long-read sequencing via Oxford Nanopore on circular artificial mini-chromosomes (42–52 kb) derived from yeast using the NucleoBond™ HMW DNA Kit and on bacteria-derived artificial mini-chromosomes prepared with the Easy-C protocol (Fig. 3). Artificial mini-chromosomes extracted from yeast could not be visualized after restriction digestion, and sequencing yielded low read counts (~66) with poor-quality data. In contrast, bacteria-derived artificial mini-chromosomes recovered using Easy-C displayed the expected digestion patterns, were successfully linearized and visualized, and produced high-quality Nanopore sequencing data with a high read count (~2000). Circular DNA typically yields low Nanopore read counts, whereas linearized constructs sequence much more efficiently. Linearizing artificial mini-chromosomes directly from yeast is not practical because the extraction copurifies the entire 12 Mb yeast genome, and the low-copy artificial mini-chromosomes are present at extremely low levels—undetectable by restriction digestion or gel electrophoresis—and would require additional clean-up, resulting in further DNA loss. Accordingly, the NucleoBond protocol does not recommend linearization [37]. One major advantage of Easy-C is its ability to linearize circular constructs prior to sequencing, allowing visualization of the correct pattern on agarose gels and substantially improving the sequencing depth and read quality. Artificial mini-chromosomes maintained on shuttle vectors with dual replication origins can propagate in both yeast and *E. coli*, enabling this bacteria-based workflow. In our hands, linearization significantly improved nanopore sequencing yield and quality. Poor sequencing performance from yeast-derived artificial mini-chromosomes likely results from low DNA concentration, the presence of sheared or denatured artificial mini-chromosomes, and contamination with yeast genomic DNA. These findings demonstrate that the Easy-C protocol provides a reliable and scalable method for extracting low-copy synthetic artificial mini-chromosomes from the Sc2.0 yeast strain. The superior sequencing results from the bacteria-purified constructs highlight a practical workflow for validating synthetic chromosome designs, which is essential for ensuring the accurate *in vivo* assembly of synthetic artificial mini-chromosomes and other genetic devices in synthetic genomics.

### Plasmids extracted from various species of NCYs

We performed long-read sequencing via Oxford Nanopore on 800 ng of pure plasmids derived from bacteria (Plasmidsaurus, United Kingdom), and mapped the reads against the reference genome and *de novo* assembly of the reads to rule out any recombination or mutations events (SNPs or indels). The sequence data were of high quality, and we obtained an average coverage of 450× (Fig. 4). Impressively, the sequencing results yielded high-quality data with no detectable point mutations in 95% of the candidates and confirmed the structural and sequence integrity of the recovered plasmids. Although recovering plasmids from NCYs is more challenging—likely due to differences in strain-specific cell wall characteristics, plasmid size, and stability—our results demonstrate that the EASY-C protocol is versatile and robust across phylogenetically diverse yeast species. This work will support synthetic biology projects not only in traditional hosts such as wild-type *S. cerevisiae* and *Sc2.0* synthetic strains, but also in NCY hosts. It will lay the groundwork for expanding genetic engineering tools beyond the boundaries of model organisms.

## Conclusions

The EASY-C protocol described in this study provides a robust, streamlined, and highly efficient approach for isolating plasmid DNA from yeast, using as little as 1 ml of overnight culture. It enables the successful recovery of both low- and high-copy-number plasmids from *S. cerevisiae* BY4741, as well as a 42–52-kb artificial mini-chromosome integrated into the synthetic yeast strain synIII (Sc2.0) via a low-copy pRS backbone. Notably, this method also proved effective across a wide spectrum of nonconventional yeast species, particularly for high-copy plasmids of medium length (~10–6.5 kb), showcasing the protocol’s versatility and broad applicability. EASY-C stands out for being simple to perform, cost-effective—requiring minimal reagents or equipment—and compatible with any standard commercial bacterial plasmid extraction kits. Beyond ease of use, the plasmids obtained are immediately ready for downstream molecular biology applications, including PCR amplification, restriction enzyme digestion, and nanopore-based long-read sequencing. The sequencing data derived from EASY-C-purified plasmids displayed superior quality and higher coverage compared to plasmids sequenced directly from yeast lysates using long-read nanopore sequencing analysis, making this method highly advantageous for precise and comprehensive genetic analysis. The Easy-C protocol was very efficient at extracting small-sized plasmids from *K. naganishii* and *K. aerobia*, while the protocol showed lower efficiency in *S. bombicola*, *S. batistae*, and *M. bulderi CBS8639,* probably due to the larger plasmid size used in these species. Differences in the cell wall structure of these NCYs and partial shearing of the genomic DNA during extraction could also be factors affecting the bacterial transformation efficiency. In summary, the EASY-C protocol provides a scale-down, cost- and time-efficient framework for the recovery of plasmids and artificial mini-chromosomes across diverse yeast backgrounds. By enabling high-quality DNA yields from even a modest number of transformed colonies, EASY-C simplifies and accelerates downstream molecular analyses. This approach has the potential to speed up synthetic biology workflows and facilitate more efficient genome engineering in both synthetic and nonconventional yeasts, supporting broader applications in biotechnology and industrial microbiology.

## Supplementary Material

Supplementary_Material_ysag002

Supplementary_Captions_ysag002

## Data Availability

All the experimental data generated in this paper are available either within the paper or in SYNBIO online. The sequences of pRS415, CRISPR/Cas9 creepy, and pUDbzB-41 plasmids are available in the literature [26, 27, 38]. The pRS harbouring mini-chromosome CEN/ARS and the pRS harbouring pan/ARS are constructs that are part of a manuscript in preparation and hence the sequences will be made available separately at a later stage.
