# Supplementary material for: EASY-C: Extraction and Analysis of Small Yeast Chromosomes—A rapid and universal platform for recovering artificial mini-chromosomes from synthetic Sc2.0 yeast and large plasmids from Saccharomyces cerevisiae and nonconventional yeast species
Source: Synth Biol (Oxf). 2026 Jan 21;11(1):ysag002. doi: 10.1093/synbio/ysag002 (PMC12922780; doi:10.1093/synbio/ysag002)
Supplement: Supplementary_Material_ysag002 [file supplementary_material_ysag002.pptx]

## Slide 1
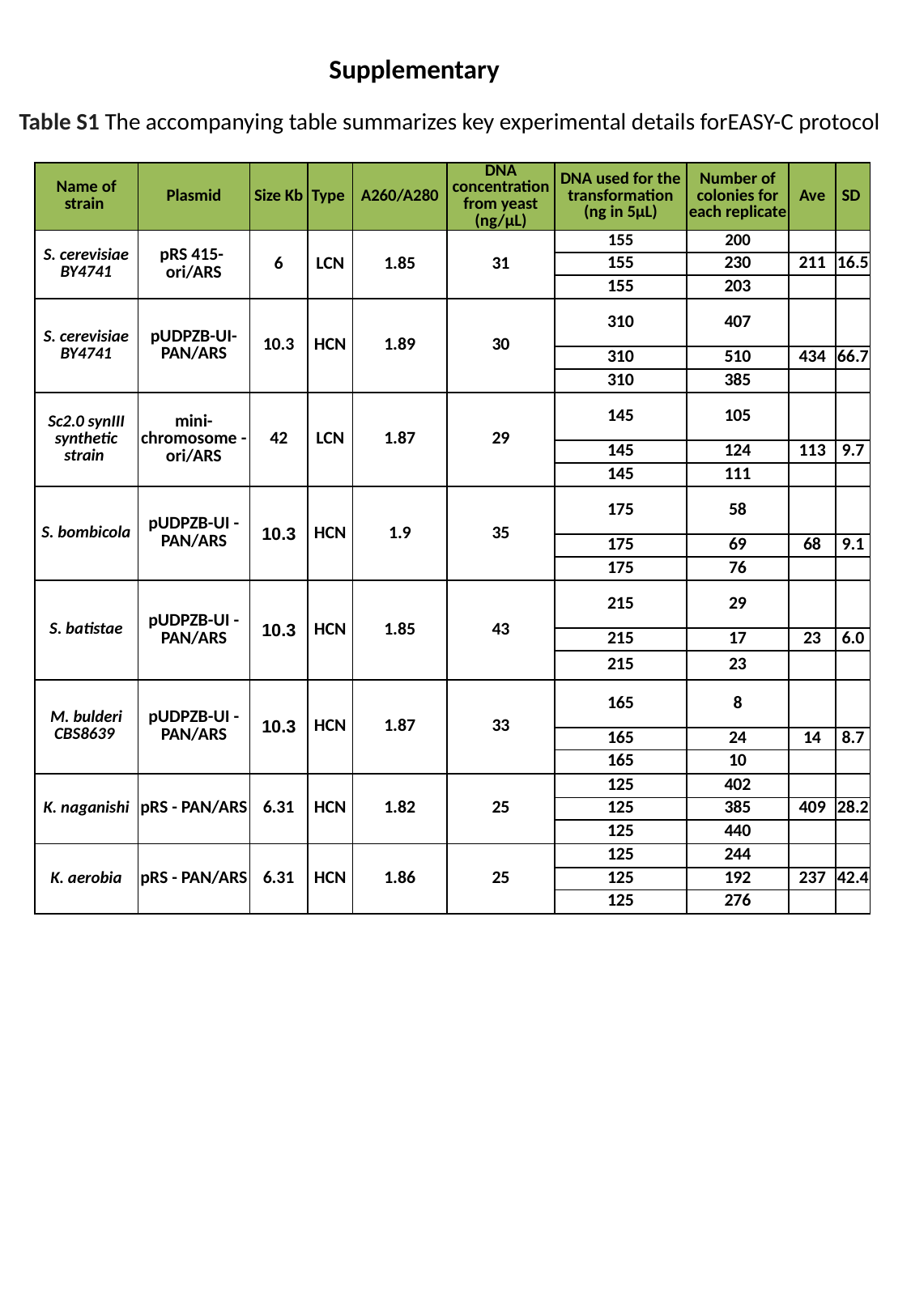

Supplementary
Table S1 The accompanying table summarizes key experimental details forEASY-C protocol
| Name of strain | Plasmid | Size Kb | Type | A260/A280 | DNA concentration from yeast (ng/µL) | DNA used for the transformation (ng in 5µL) | Number of colonies for each replicate | Ave | SD |
| --- | --- | --- | --- | --- | --- | --- | --- | --- | --- |
| S. cerevisiae BY4741 | pRS 415-  ori/ARS | 6 | LCN | 1.85 | 31 | 155 | 200 | | |
| | | | | | | 155 | 230 | 211 | 16.5 |
| | | | | | | 155 | 203 | | |
| S. cerevisiae BY4741 | pUDPZB-UI- PAN/ARS | 10.3 | HCN | 1.89 | 30 | 310 | 407 | | |
| | | | | | | 310 | 510 | 434 | 66.7 |
| | | | | | | 310 | 385 | | |
| Sc2.0 synIII synthetic strain | mini-chromosome - ori/ARS | 42 | LCN | 1.87 | 29 | 145 | 105 | | |
| | | | | | | 145 | 124 | 113 | 9.7 |
| | | | | | | 145 | 111 | | |
| S. bombicola | pUDPZB-UI -PAN/ARS | 10.3 | HCN | 1.9 | 35 | 175 | 58 | | |
| | | | | | | 175 | 69 | 68 | 9.1 |
| | | | | | | 175 | 76 | | |
| S. batistae | pUDPZB-UI -PAN/ARS | 10.3 | HCN | 1.85 | 43 | 215 | 29 | | |
| | | | | | | 215 | 17 | 23 | 6.0 |
| | | | | | | 215 | 23 | | |
| M. bulderi CBS8639 | pUDPZB-UI -PAN/ARS | 10.3 | HCN | 1.87 | 33 | 165 | 8 | | |
| | | | | | | 165 | 24 | 14 | 8.7 |
| | | | | | | 165 | 10 | | |
| K. naganishi | pRS - PAN/ARS | 6.31 | HCN | 1.82 | 25 | 125 | 402 | | |
| | | | | | | 125 | 385 | 409 | 28.2 |
| | | | | | | 125 | 440 | | |
| K. aerobia | pRS - PAN/ARS | 6.31 | HCN | 1.86 | 25 | 125 | 244 | | |
| | | | | | | 125 | 192 | 237 | 42.4 |
| | | | | | | 125 | 276 | | |

## Slide 2
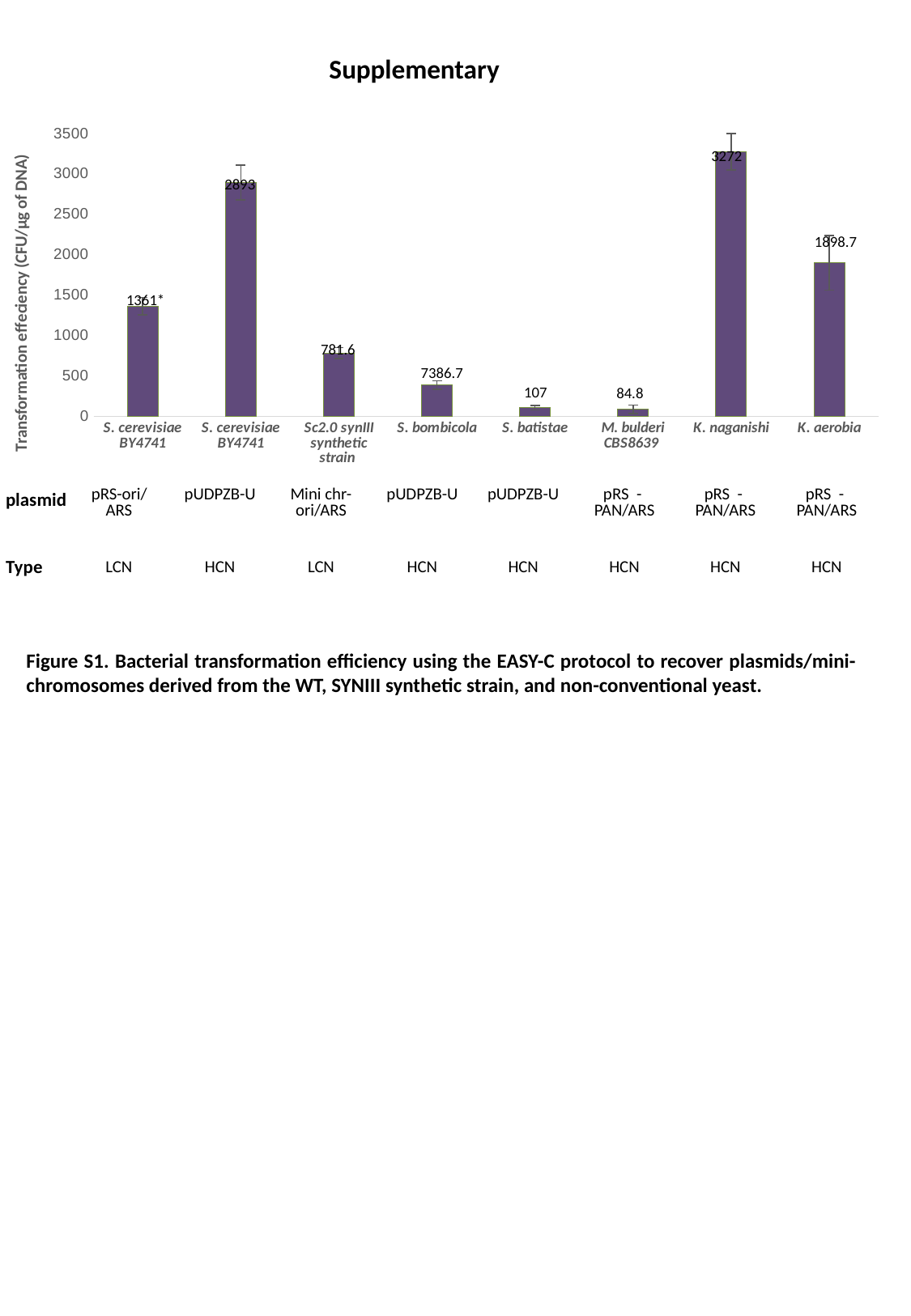

Supplementary
### Chart
| Category | |
|---|---|
| S. cerevisiae BY4741
 | 1361.2903225806451 |
| S. cerevisiae BY4741
 | 2893.0 |
| Sc2.0 synIII synthetic strain
 | 781.609195402299 |
| S. bombicola | 386.6666666666667 |
| S. batistae | 106.97674418604652 |
| M. bulderi CBS8639  | 84.84848484848486 |
| K. naganishi | 3272.0 |
| K. aerobia | 1898.6666666666667 |3272
2893
1898.7
1361*
781.6
7386.7
107
84.8
| pRS-ori/ARS | pUDPZB-U | Mini chr-ori/ARS | pUDPZB-U | pUDPZB-U | pRS  -  PAN/ARS | pRS  -  PAN/ARS | pRS  -  PAN/ARS |
| --- | --- | --- | --- | --- | --- | --- | --- |
| LCN | HCN | LCN | HCN | HCN | HCN | HCN | HCN |
plasmid
Type
Figure S1. Bacterial transformation efficiency using the EASY-C protocol to recover plasmids/mini-chromosomes derived from the WT, SYNIII synthetic strain, and non-conventional yeast.

## Slide 3
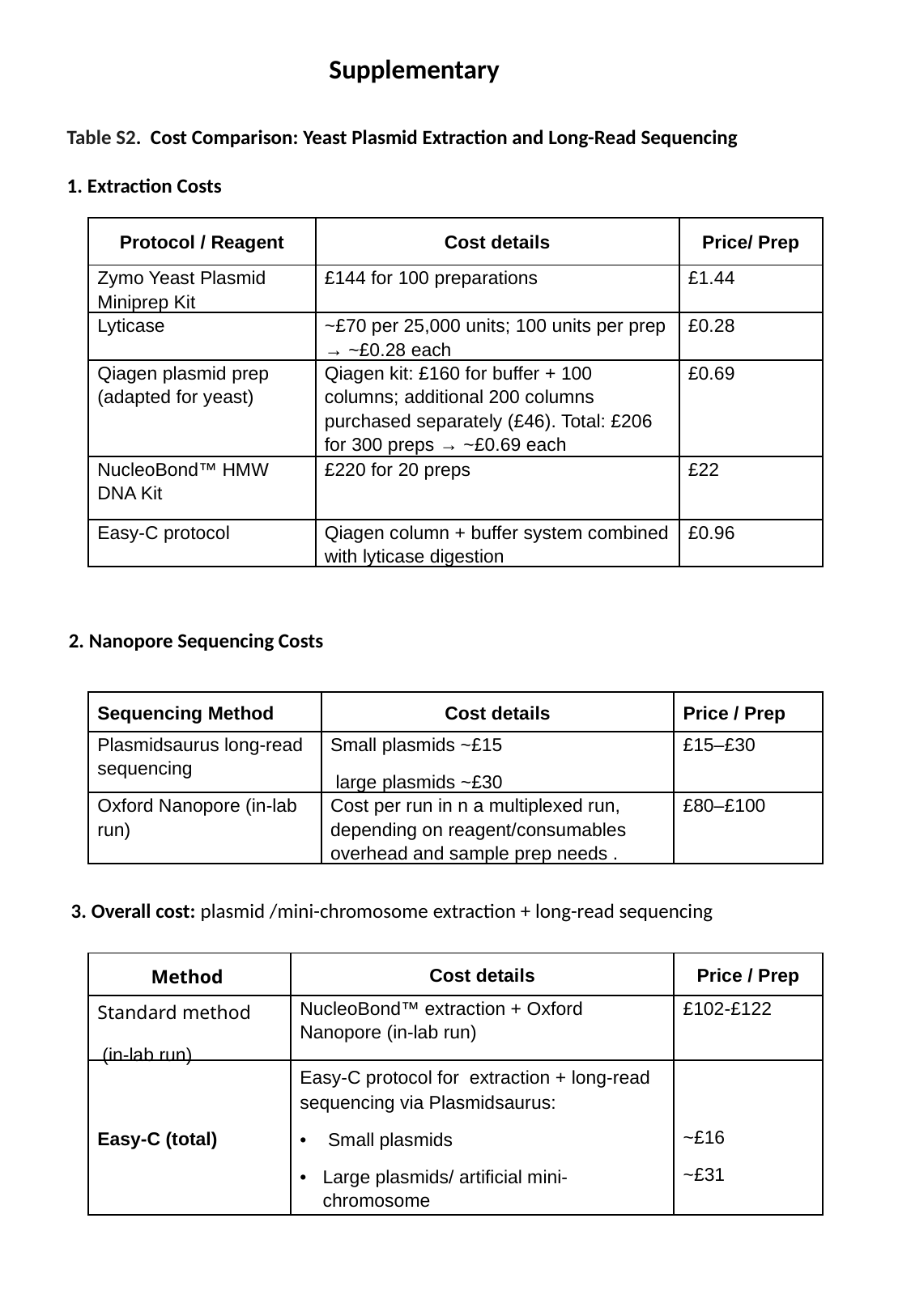

Supplementary
Table S2. Cost Comparison: Yeast Plasmid Extraction and Long-Read Sequencing
1. Extraction Costs
| Protocol / Reagent | Cost details | Price/ Prep |
| --- | --- | --- |
| Zymo Yeast Plasmid Miniprep Kit | £144 for 100 preparations | £1.44 |
| Lyticase | ~£70 per 25,000 units; 100 units per prep → ~£0.28 each | £0.28 |
| Qiagen plasmid prep (adapted for yeast) | Qiagen kit: £160 for buffer + 100 columns; additional 200 columns purchased separately (£46). Total: £206 for 300 preps → ~£0.69 each | £0.69 |
| NucleoBond™ HMW DNA Kit | £220 for 20 preps | £22 |
| Easy-C protocol | Qiagen column + buffer system combined with lyticase digestion | £0.96 |
2. Nanopore Sequencing Costs
| Sequencing Method | Cost details | Price / Prep |
| --- | --- | --- |
| Plasmidsaurus long-read sequencing | Small plasmids ~£15 large plasmids ~£30 | £15–£30 |
| Oxford Nanopore (in-lab run) | Cost per run in n a multiplexed run, depending on reagent/consumables overhead and sample prep needs . | £80–£100 |
3. Overall cost: plasmid /mini-chromosome extraction + long-read sequencing
| Method | Cost details | Price / Prep |
| --- | --- | --- |
| Standard method (in-lab run) | NucleoBond™ extraction + Oxford Nanopore (in-lab run) | £102-£122 |
| Easy-C (total) | Easy-C protocol for extraction + long-read sequencing via Plasmidsaurus: Small plasmids Large plasmids/ artificial mini-chromosome | ~£16 ~£31 |

## Slide 4
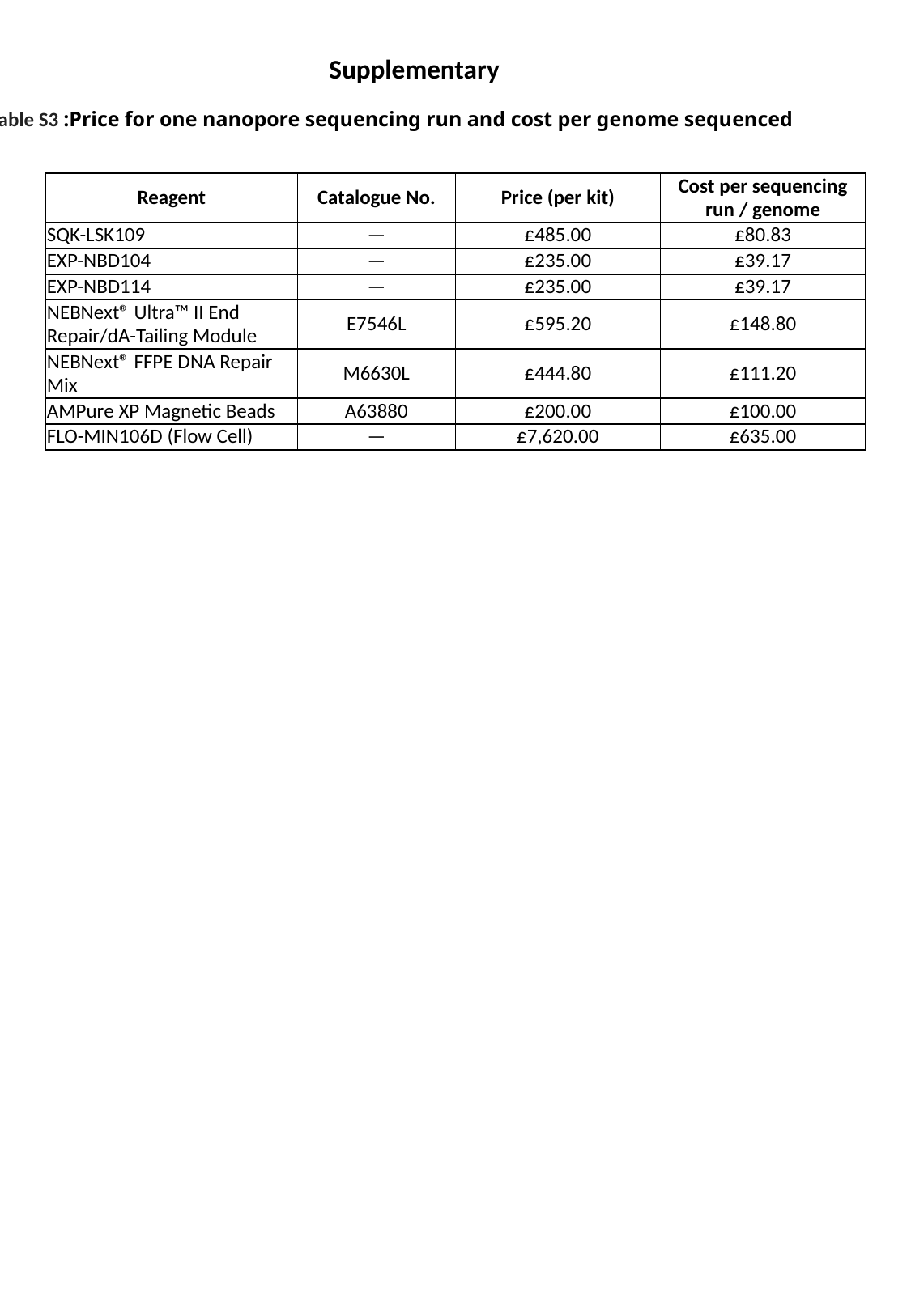

Supplementary
Table S3 :Price for one nanopore sequencing run and cost per genome sequenced
| Reagent | Catalogue No. | Price (per kit) | Cost per sequencing run / genome |
| --- | --- | --- | --- |
| SQK-LSK109 | — | £485.00 | £80.83 |
| EXP-NBD104 | — | £235.00 | £39.17 |
| EXP-NBD114 | — | £235.00 | £39.17 |
| NEBNext® Ultra™ II End Repair/dA-Tailing Module | E7546L | £595.20 | £148.80 |
| NEBNext® FFPE DNA Repair Mix | M6630L | £444.80 | £111.20 |
| AMPure XP Magnetic Beads | A63880 | £200.00 | £100.00 |
| FLO-MIN106D (Flow Cell) | — | £7,620.00 | £635.00 |
