# Supplementary material for: EASY-C: Extraction and Analysis of Small Yeast Chromosomes—A rapid and universal platform for recovering artificial mini-chromosomes from synthetic Sc2.0 yeast and large plasmids from Saccharomyces cerevisiae and nonconventional yeast species
Source: Synth Biol (Oxf). 2026 Jan 21;11(1):ysag002. doi: 10.1093/synbio/ysag002 (PMC12922780; doi:10.1093/synbio/ysag002)
Supplement: Supplementary_Captions_ysag002 [file supplementary_captions_ysag002.docx]

**Supplementary**

**Table S1.** **The accompanying table summarizes key experimental details,** including the yeast species used, plasmid names, copy number type (high or low), plasmid size, A260/A280 ratio, DNA yield (from 1 mL overnight culture), and the number of *E. coli* *DH5α* transformants. HCN: high copy number; LCN: low copy number. Standard deviations were calculated from three replicates. Bacterial transformation efficiency is shown in Fig.S 1. Plasmids (1–3) derived from bacteria were subjected to nanopore sequencing.

**Table S2. Cost Comparison: Yeast Plasmid Extraction and Long-Read Sequencing**

**Figure S1. Bacterial transformation efficiency using the EASY-C protocol to recover plasmids/artificial mini-chromosomes derived from the WT, SYNIII synthetic strain, and non-conventional yeast.**

The figure shows transformation efficiency as CFU per µg of circular DNA. HCN: high copy number; LCN: low copy number. * Mean number of colonies from three biological replicates, with error bars representing ± SEM Please note that the circular DNA recovered from yeast may contain sheared circular DNA and genomic DNA contamination. Therefore, the CFU per µg of DNA is expected to be lower compared to transformations using pure supercoiled circular DNA recovered from bacteria.
